# Demographic and clinical characteristics of children and adolescents hospitalised with laboratory-confirmed COVID-19 in the Tel-Aviv District, Israel, 2020–2022

**DOI:** 10.1017/S0950268823000250

**Published:** 2023-02-21

**Authors:** Matanelle Salama, Ziva Amitai, Rivka Sheffer

**Affiliations:** Tel Aviv District Health Office, Ministry of Health, Tel Aviv, Israel

**Keywords:** COVID-19, infectious disease epidemiology, vaccine preventable diseases

## Abstract

Our study population consisted of all children and adolescents, with laboratory-confirmed SARS-Co-V-2 infection, hospitalised from February 2020 through February 2022, among residents of the Tel Aviv (TA) District, Israel. There were 491 children and adolescents hospitalised with Sars-CoV-2 infection. Among them, 281 (57%) admitted with coronavirus disease 2019 (COVID-19) as the primary cause of admission (rate of 39 per 100 000). Among all children and adolescents in the TA District, the highest hospitalisation rates were observed among infants and children below the age of 4 years (rate of 311 per 100 000 population). Severe disease was observed mostly among children with multiple underlying medical conditions. Admission rates were also elevated among residents of the ultra-orthodox community (rate ratio (RR) compared to the rest of the district; 95% confidence interval (CI) 2.38–3.82). Admission rates with COVID-19 as primary cause of admission were higher during Omicron compared to Delta predominance period (RR 1.7; 95% CI 1.22–2.32). Targeted social and public health policies should be put in place when rates of disease start to increase, such as encouraging vaccine uptake for eligible children and social distancing when necessary, taking into account already existing social and learning gaps, in order to reduce the burden of disease.

## Introduction

Children, up to 18 years of age, have been an integral part of the pandemic, in terms of their susceptibility [1] and infectiousness [2, 3]. Although less commonly than in adults, severe outcomes, as a result of COVID-19, have also been shown to occur among children and adolescents [4].

A mass vaccination campaign with the Pfizer-BioNTech coronavirus vaccine was launched in Israel in December 2020, initially for adults and adolescents over 16 years of age, and subsequently expanded to include children over 5 years of age [5].

Because of ongoing waves, a third booster dose of the mRNA vaccine was recommended to all adults during the SARS-CoV-2 B.1.617.2 (Delta) wave, and a fourth booster dose was recommended to at-risk populations during SARS-CoV-2 B.1.1.529 (Omicron) predominance [6].

This population-based study is the first of our knowledge to capture all admissions from children living in a single residential area (in our study, the Tel Aviv (TA) District, Israel), giving a clear estimate of the disease burden. We aim to provide a benchmark of the epidemiologic and clinical characteristics of children hospitalised with COVID-19 in the TA District, Israel, from the start of the pandemic, and to compare our findings between the Delta and Omicron predominance periods.

## Methods

### Study population and data collection

Our study population consisted of children and adolescents, up to 18 years of age, residing in the TA District, Israel and hospitalised with COVID-19, from 1 February 2020 to 28 February 2022.

The TA District is comprised of 12 cities located on the western coastline of Israel and has a population of approximately 1.5 million people, among them 398 857 (27%) are under 18 years of age. The district is known for its diverse ethnic population, which is largely defined by city of residence. The city of Bnei Brak is the most densely populated city in the district (and in the country), with a predominantly ultra-orthodox Jewish population (92% of its residents), with high birth rates (crude birth rate of 38.4 per 1000 population) and low socio-economic status.

COVID-19 became a reportable disease by law in Israel in February 2020, and all verified COVID-19 results are automatically entered in a national database whenever they test positive for Sars-CoV-2. This database was instated for surveillance purposes by the Ministry of Health (MOH) Data Monitoring Committee, and includes data on hospitalised patients. Data on individual patients were extracted from personal hospital files, available as part of the disease notification mandate. It gives legal authority to analyse the recorded data and to report it. For the purpose of this study, all patients from this existing database were de-identified.

All cases in the study were confirmed by real-time polymerase chain reaction during their hospitalisation or during the 14 days prior to hospital admission. Patients with a positive antigen detection rapid diagnostic test were given a PCR test for confirmation at the time of admission.

Population estimates were extracted from the Israeli Central Bureau of Statistics [7].

Vaccination campaign with the Pfizer-BioNTech mRNA vaccine was available for adolescents aged 16 years and above from 20 December 2020; for adolescents aged 12–15 years from July 2021, for children aged 5–11 years from the end of October 2021 [8]. All vaccines were given free of charge for all residents.

### Variant predominance period definition

Delta predominance period was determined to be from 15 June 2021 to 15 December 2021 and Omicron predominance period was set from 16 December 2021 until the end of the study period 28 February 2022, based on routine laboratory surveillance (MoH, unpublished data).

### Analysis

We describe the number and rates of hospital admissions of children and adolescents with laboratory-confirmed COVID-19 infections in the TA District, Israel, and compare these rates by Delta and Omicron predominance periods.

Our secondary analysis was among the admissions with COVID-19 as primary cause of hospitalisation. For these admissions, we describe demographic, vaccination status as well as clinical outcomes including length of stay, disease severity as indicated by admissions to the ICU, diagnosis of multisystem inflammatory syndrome (MIS-C) and mortality.

Lastly, we conduct a subgroup analysis among the group of interest with Bnei Brak as their primary city of residence.

## Results

### Epidemiologic characteristics

**Fig. 1.**
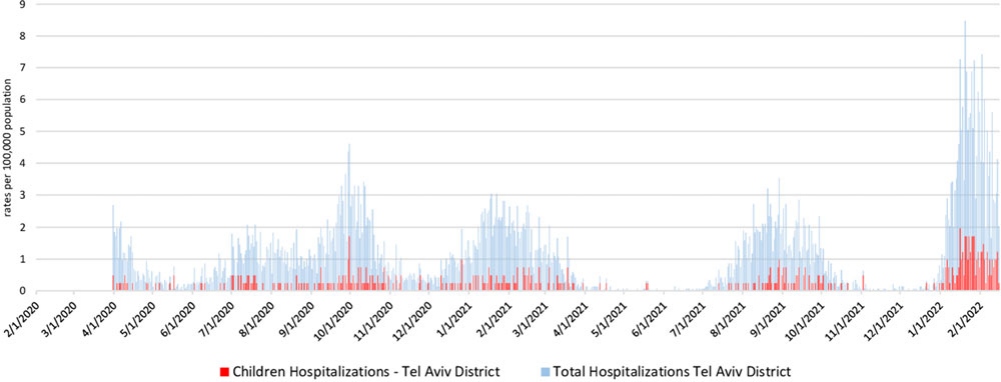
Total and children and adolescent COVID-19 hospitalisation rates in the Tel Aviv District, Israel, 2020–2022.

From February 2020 to 28 February 2022, there were 64 683 reported hospitalisations with positive Sars-CoV-2 infection in Israel. Among them, 3810 (6%) were children. In the TA District, during that same period, there were 9414 reported admissions with COVID-19, among them 491 were children, accounting for 5% of total COVID-19 hospitalisations (Fig. 1). Among all children and adolescent hospitalisations in the TA District, 281 were hospitalised with COVID-19 as primary cause of admission. Among them, 188 (67%) children below the age of 4 years (rate of 311 per 100 000 population).

### Clinical presentation

Among all 491 eligible admissions, complete clinical data were available for 478 (96%) cases: 281 (57%) patients were admitted with COVID-19 as their cause of hospitalisation.

Among the 281 patients admitted with COVID as their primary cause of hospitalisation: 165 (59%) patients were under the age of 1 year, 23 (8%) were between 2 and 4 years, 36 (13%) were between 5 and 11 years and 57 (20%) were between 12 and 18 years. Overall, 123 (44%) patients were admitted with fever as their sole symptom. Other clinical presentations included upper respiratory symptoms among 124 (44%) patients, gastro-intestinal symptoms among 52 (19%) patients, pneumonia among 26 (9%) patients, myocarditis and/or pericarditis among three (1%) patients and MIS-C among 15 (5%) patients. One or more underlying medical conditions were present among 76 (27%) patients.

Median length of stay was 3 days (range 1–34 days). Hospitalisations in the ICU took place for 28 (10%) patients, among them nine (33%) infants with neonatal fever were admitted for observation purposes, eight (30%) patients with MIS-C, 10 (22%) patients required respiratory assistance due to their dyspnoea and oxygen desaturation (three with underlying respiratory conditions, seven with underlying neurologic/developmental condition and one premature infant), and one patient (with underlying immune deficiency) was admitted for observation purposes because of chest pain, without any findings suggesting the presence of myocarditis or pericarditis.

There were three (1%) deaths as a result of COVID-19 in our study: one 12-year-old patient as a result of MIS-C, without any underlying condition; and two 16-year-old patients with multiple underlying conditions. All of those cases were unvaccinated against coronavirus.

During the entire study period, among 101 vaccine-eligible patients, five (5%) were vaccinated. Among the 35 vaccine-eligible adolescents aged above 16 years, three were fully vaccinated. Among the 29 vaccine-eligible patients aged 12–15 years, one patient was vaccinated (this patient was immune suppressed and, prior to their hospitalisation, had received one dose of vaccine after having recovered from a previous infection with Sars-coV-2). Among 37 vaccine-eligible patients aged 5–11 years, one was fully vaccinated.

During Delta predominance, 59 (69%) children were hospitalised because of their COVID-19 *vs.* 69 (59%) children during Omicron predominance (*P* = 0.1). Other causes of hospitalisations during Delta and Omicron predominance periods are presented in Table 1.

**Table 1.** Clinical characteristics of hospitalised patients with COVID-19 in the Tel Aviv District, Israel, during Delta and Omicron predominance periods, 2021–2022 (*N* = 254)

Variant predominance period	Total (*n* = 254)	Delta (B.1.617.2) predominance *N* = 85	Omicron (B.1.1.529) predominance *N* = 169	*P* value
Age group				
0–1	122 (48%)	35 (41%)	87 (52%)	0.1
2–4	28 (11%)	8 (9%)	20 (12%)	0.6
5–11	58 (23%)	22 (26%)	36 (21%)	0.4
12–18	46 (18%)	20 (24%)	26 (15%)	0.1
Gender				
Male	143 (56%)	48 (57%)	95 (56%)	0.9
Female	111 (44%)	37 (44%)	74 (44%)	0.9
Cause of hospitalisation				
Hospitalised because of COVID-19	158 (62%)	59 (69%)	99 (59%)	0.1
Trauma/surgery	35 (14%)	9 (11%)	26 (15%)	0.3
Obstetrics/gynaecology	1 (0%)	1 (1%)	0	0.2
Psychiatry	3 (1%)	1 (1%)	2 (1%)	0.9
Surveillance/quarantine purposes	5 (2%)	2 (2%)	3 (2%)	0.7
Seizures/febrile seizures	15 (6%)	3 (4%)	12 (7%)	0.3
Oncology/haematology	9 (4%)	1 (1%)	8 (5%)	0.2
Other	18 (7%)	6 (7%)	12 (7%)	0.9
Unknown	10 (4%)	3 (4%)	7 (4%)	0.4
Median length of stay (range)	3 (1-30)	3 (1–10)	3 (1-30)	0.3
Symptoms				
Fever (only)	51 (20%)	19 (22%)	32 (19%)	0.5
Upper respiratory	82 (32%)	23 (27%)	59 (35%)	0.2
Gastro-intestinal	38 (15%)	66 (78%)	19 (11%)	0.02
Pneumonia	16 (6%)	6 (7%)	10 (6%)	0.7
Seizures/febrile seizures	6 (2%)	2 (2%)	5 (3%)	0.9
Myocarditis/pericarditis	2 (1%)	1 (1%)	1 (1%)	0.6
MIS-C	11 (4%)	11 (13%)	0	0.4
Underlying condition				
Developmental/neurologic disorder	18 (7%)	4 (5%)	14 (8%)	0.3
Underlying respiratory conditions	21 (8%)	10 (12%)	11 (7%)	0.2
Epilepsy/seizures	13 (5%)	4 (5%)	9 (5%)	0.9
Oncology/haematology	19 (7%)	2 (2%)	14 (8%)	0.07
Underlying liver/gastro-intestinal condition	10 (x)	3 (4%)	6 (4%)	0.9
Underlying heart condition	5 (2%)	1 (1%)	4 (2%)	0.5
Death	2 (1%)	0	2 (1%)	0.3
ICU stay	16 (6%)	7 (8%)	9 (5%)	0.4
Co-occurring respiratory infection				
RSV	10(4%)	0	10 (6%)	0.02
Adenovirus	3 (1%)	2 (2%)	1 (1%)	0.2
Rhinovirus	1	1 (1%)	2 (1%)	0.9
Influenza	1	1 (1%)	0	0.2
Co-occurring UTI	4 (2%)	3 (4%)	1 (1%)	0.2
Ultra-orthodox city of Bnei Brak	83 (33%)	28 (33%)	55 (33%)	0.9

When comparing Delta and Omicron prevalence periods among all hospitalized children and adolescents with regard to their COVID-19 symptoms, clinical presentations were similar, except for gastro-intestinal symptoms which were significantly more present during the Delta period (78% *vs.* 11%, respectively). Admission to the ICU occurred among seven (8%) patients during Delta predominance and nine (5%) patients during Omicron predominance. Co-occurring infections with respiratory syncytial virus (RSV) were present during Omicron predominance period only (Table 1).

### Comparison between waves

In the TA District, during Delta predominance, 32 927 children and adolescents tested positive for Sars-CoV-2 (rate of 8099 per 100 000 population) and 85 (20.9 per 100 000 population) were hospitalised with Sars-CoV-2 infection. During Omicron, 111 538 children and adolescents tested positive for Coronavirus (27 436 per 100 000 population) and 169 (41.57 per 100 000 population) were hospitalised with Sars-CoV-2 infection (rate ratio (RR) for hospitalisation between Delta and Omicron 1.99, 95% CI 1.53–2.58). Of note, reported numbers of positive tests for adults were 44 952 (3095 per 100 000 population) during Delta predominance and 233 074 (16 047 per 100 000 population) during Omicron predominance. Overall adult hospitalisation rates during Delta and Omicron predominance periods were 572.43 and 602.36 per 100 000 population, respectively (RR 1.05, 95% CI 1–1.1).

Among children and adolescents hospitalised because of COVID-19, rates of hospitalisations for children aged 0–1 year were 50.94 per 100 000 during Delta predominance and 124.52 per 100 000 during Omicron (RR 2.44; 95% CI 1.57–3.83); rates for children aged 2–4 years were 5.29 per 100 000 during Delta predominance and 17.2 during Omicron predominance (RR 3.25; 95% CI 1.06–9.97) (Table 2).

**Table 2.** Rates and rate ratios of children and adolescents hospitalisations with COVID-19 as a primary reason of admission during Delta and Omicron predominance periods, in the Tel Aviv District, Israel, 2020–2022 (*N* = 158)

	Delta predominance	Omicron predominance	Rate ratio (95% CI)
0–1 year	50.94 per 100 000	124.52 per 100 000	2.44 (1.57–3.83)
2–4 years	5.29 per 100 000	17.2 per 100 000	3.25 (1.06–9.97)
5–11 years	9.1 per 100 000	7.8 per 100 000	0.86 (0.4–1.9)
12–18 years	11.3 per 100 000	6.45 per 100 000	0.68 (0.24–1.36)
Total	14.51 per 100 000	24.35 per 100 000	1.68 (1.22–2.32)

### Subgroup analysis

Overall cumulative hospitalisation rates in children and adolescents were 207.6 per 100 000 population in Bnei Brak and 90.53 per 100 000 in the rest of the district (RR 2.29; CI 2.59–3.69).

Among the 281 patients (rate of 69 per 100 000 children and adolescents in the TA District) hospitalised because of their COVID-19, 123 (44%) were residents of the ultra-orthodox city of Bnei Brak (rate of 117 per 100 000 children and adolescents in Bnei Brak). Hospitalisation rates with COVID-19 as the primary reason of hospitalisation were elevated across all age categories in Bnei Brak compared to the rest of the district, specifically for children aged 0–4 years (Table 3). Among those patients, 28 (10%) had one or more underlying medical condition and seven (3%) required treatment in the ICU. One patient died as a result of COVID-19 in this group.

**Table 3.** Rates and rate ratios of children and adolescent hospitalisations with COVID-19 as primary reason of admission in Bnei Brak and in the Tel Aviv District (not including Bnei Brak), Israel, 2020–2022 (*N* = 281)

	Bnei Brak	Tel Aviv District, not including Bnei Brak	Rate ratio (95% CI)
0–1 year	516.43 per 100 000	166.03 per 100 000	3.11 (2.29–4.22)
2–4 years	59.21 per 100 000	14.56 per 100 000	4.07 (1.79–9.22)
5–11 years	28.44 per 100 000	16.24 per 100 000	1.75 (0.87–3.56)
12–18 years	73.87 per 100 000	27.39 per 100 000	2.7 (1.59–4.58)
Total	117.15 per 100 000	38.87 per 100 000	3.01 (2.38–3.82)

During both Delta and Omicron predominance, 33% of hospitalised patients and adolescents were residents of the city of Bnei Brak (Table 1).

In Bnei Brak, during Delta predominance period, 8400 (rate of 8000.38 per 100 000) children and adolescents tested positive for Sars-CoV-2 and 9259 (rate of 8818.52 per 100 000) children and adolescents during Omicron predominance period (26% *vs.* 8% respectively of total positive tests among children and adolescents in the TA District as a whole).

## Discussion

This study analyses the characteristics of reported hospital admissions with COVID-19 among children and adolescents in the TA District, Israel, from February 2020 through February 2022, both Delta and Omicron wave inclusive. In our study, thrice as many children and adolescents tested positive for coronavirus during Omicron compared to Delta predominance (which is probably an underestimate as many cases were diagnosed with at home antigen tests which were not always reported to the MOH). Hospital admission rates among children and adolescents were twice as high during Omicron compared to Delta predominance period (42 *vs.* 21 per 100 000 population), which were lower compared to surveillance reports from other states [9]. In contrast, there was a fivefold increase in the number of adults who tested positive for Sars-CoV-2 infection between the two variant predominance periods but the number of adult hospitalisations was similar during both waves, with a slight increase during Omicron compared to Delta. As a result, the proportion of hospitalisations among positive patients was much higher during Delta compared to Omicron predominance among adults, probably owing to the administration of COVID-19 vaccine booster doses, before the onset of the Omicron wave.

The proportion of admissions with COVID-19 as cause of hospitalisation was similar between the two variant predominance waves among children and adolescents, which might have been due to previous exposures that imparted immunity as well as vaccinations among adolescents.

It is worth noting that we did not have molecular sequencing analysis for each of our hospitalised patients and some cases might have been hospitalised with the Delta variant during the Omicron prevalence period. This might have led to an overestimation of Omicron prevalence.

The Delta predominance period occurred mostly during summer break and the month of September, throughout most of which schools were closed because of the Jewish High Holidays. In contrast, schools stayed open during the entirety of the Omicron predominance period. Mitigation measures put in place during that period in schools included the distribution of home antigen tests that were to be administered to students twice a week as well as mandatory masking for students aged above 6 years and school staff. Students who tested positive on their home antigen test were to isolate for 5 days, their class contacts were allowed to attend school with no limitations.

Two deaths as a result of COVID-19 were observed during Omicron predominance period whereas none were observed during Delta predominance. Both deaths were among children with several severe underlying conditions, and both were eligible for a COVID-19 vaccine but had not been vaccinated.

Out of 101 vaccine-eligible patients during the course of this study, five (5%) had been fully or partially vaccinated. Adherence to COVID vaccination recommendations for children and adolescents in Israel was unusually low (approximately 20% fully vaccinated children and adolescents with the mRNA vaccine, MOH, unpublished data), as compliance to routine vaccine recommendations are typically very high (93% of children fully vaccinated for routine vaccinations in the TA District, MOH – unpublished data). This might have been caused by widespread discussions in the general public on vaccinations that elicited doubts and vaccine hesitancy.

Children under the age of 4 years, and more specifically under the age of 1 year, were the most vulnerable to hospitalisation and were not eligible for vaccination during the study period. However, their clinical presentation was mostly mild with fever as the only symptom (48%) or upper respiratory symptoms (38%) and median length of stays of 3 days. In this age group, admissions in the ICU were mostly for observation purposes as a precautionary measure and no deaths occurred.

The highest admission rates in the TA District were observed among the ultra-orthodox community of the city of Bnei Brak, with roughly thrice the risk of hospital admission compared to the rest of the district. This is the most densely populated city in the district with 209 000 residents, including 105 000 children and adolescents (50%), with the lowest socio-economic status. Although during both Delta and Omicron predominance periods, one-third of hospitalised children and adolescents were from Bnei Brak, the proportion of children and adolescents testing positive during omicron was lower (26% during Delta *vs.* 8% during Omicron of total children tested). This possibly indicates a lower compliance to testing recommendations in Bnei Brak during Omicron, where disease rates were high, and thus hospital admission rates in that community gave us a better approximation of the burden of disease.

The most common underlying medical conditions among hospitalised children were underlying respiratory conditions, developmental or neurological disorders and haematologic or oncologic condition. It is critical to address those susceptible patients with precautionary mitigation measures during disease waves, and to encourage vaccine uptake among eligible children.

Infants and children below the age of 4 years had the highest rates of hospitalisation, as well as populations with lower socio-economic status, although their symptoms were mostly mild. Severe disease was observed mostly among children with multiple underlying medical conditions. Targeted social and public health policies be put in place when rates of disease start to increase, such as encouraging vaccine uptake for eligible children and social distancing when necessary, taking into account already existing social and learning gaps.

## Data Availability

Raw data for this study were generated in a database that was instated for surveillance purposes by the Ministry of Health (MOH) Data Monitoring Committee, and includes data on hospitalised patients. Data on individual patients were extracted from personal hospital files, and are available from M. S. on request.
